# Sister chromatid separation and monopolar spindle organization in the first meiosis as two mechanisms of unreduced gametes formation in wheat–rye hybrids

**DOI:** 10.1007/s00497-016-0279-5

**Published:** 2016-03-18

**Authors:** O. G. Silkova, D. B. Loginova

**Affiliations:** Institute of Cytology and Genetics, Siberian Branch of the Russian Academy of Sciences, Lavrentiev Ave. 10, Novosibirsk, 630090 Russia

**Keywords:** Amphihaploids, FISH, Immunostaining, Mitotic-like division, Monopolar spindle, Unreduced gametes

## Abstract

*****Key message***:**

**Unreduced gametes.**

**Abstract:**

The absence of a strict pachytene checkpoint in plants presents an opportunity to study meiosis in polyhaploid organisms. In the present study, we demonstrate that meiosis is coordinated in hybrids between disomic wheat–rye substitution lines 1Rv(1A), 2R(2D), 5R(5D), 6R(6A) and rye (*Triticum aestivum* L. × *Secale cereale* L., 4*x* = 28, ABDR). By using in situ hybridization with a centromere pAet6-09 probe and immunostaining with H3Ser10ph-, CENH3-, and α-tubulin-specific antibodies, we distinguished four chromosome behaviour types. The first one is a mitotic-like division that is characterized by mitotic centromere architecture, robust bipolar spindle, one-step loss of arm and centromere cohesion, and sister chromatid separation in the first and only meiotic division. The second type involves a monopolar spindle formation, which appears as a hat-shaped group of chromosomes moving in one direction, wherein MT bundles are co-oriented polewards. It prevents chromosome segregation in meiosis I, with a bipolar spindle distributing sister chromatids to the poles in meiosis II. These events subsequently result in the formation of unreduced microspores. The other two meiotic-like chromosome segregation patterns known as reductional and equational plus reductional represent stand-alone types of cell division rather than intermediate steps of meiosis I. Only sterile pollen is produced as a result of such meiotic-like chromosome behaviours. Slightly variable meiotic phenotypes are reproducibly observed in hybrids under different growth conditions. The 2R(2D)xR genotype tends to promote reductional division. In contrast, the genotypes 1Rv(1A)xR, 5R(5D)xR, and 6R(6A)xR promote equational chromosome segregation and monopolar spindle formation in addition to reductional and equational plus reductional division types.

**Electronic supplementary material:**

The online version of this article (doi:10.1007/s00497-016-0279-5) contains supplementary material, which is available to authorized users.

## Introduction

Polyploidy is widespread among flowering plants, and allopolyploidy is one of the major speciation pathways in plant evolution (Adams and Wendel 2005; Otto 2007; Soltis et al. 2009; Soltis and Soltis 2009; Feldman and Levy 2012; Tayale and Parisod 2013; Estep et al. 2014). Allopolyploidy pathway produces adaptive species with high genomic plasticity and capable of occupying novel ecological niches (Wendel 2000; Feldman and Levy 2005).

Allopolyploidy is intimately associated with a nascent hybridization event, as allopolyploids are formed during interspecific/intergeneric hybridization followed by chromosome doubling through the union of unreduced gametes, via somatic doubling, or by means of a triploid bridge and other mechanisms (Bretagnolle and Thompson 1995; Ramsey and Schemske 1998, 2002; Feldman and Levy 2005). Thus, related yet diverged genomes are combined in one allopolyploid genome. Bread wheat *Triticum aestivum* L. is a typical allopolyploid species. The bread wheat subgenomes A, B, and D were originally derived from three diploid (2*x*; 2*n* = 14) species within tribe Triticeae: *Triticum urartu* (AA), an extinct or yet undiscovered species from *Aegilops speltoides* (BB) lineage, and *Ae. tauschii* (DD) (IGWSC 2014). The primary allopolyploidization event involved A and B genome donors resulting in the extant tetraploid emmer wheat (*T. turgidum*; AABB); the second allopolyploidization event between emmer wheat and the D genome donor formed modern hexaploid bread wheat (AABBDD) (Feldman and Levy 2005; Petersen et al. 2006). Recently, an alternative evolutionary scenario was proposed for the bread wheat (Marcussen et al. 2014). According to this scenario, the very first hybridization event between the ancestral A and B genome lineages occurred about 5.5 MYA and led to the origin of the D genome lineage by homoploid hybrid speciation. The second hybridization event (less than 0.8 MYA) between a close relative (BB) of *Ae. speltoides* and *T. urartu* (AA) gave rise to the allotetraploid emmer wheat (*T. turgidum*; AABB) by polyploidization. Bread wheat originated by allopolyploidization from a third hybridization (less than 0.8 MYA), between emmer wheat and *Ae. tauschii* (DD). Despite being polyploid, bread wheat displays diploid-like meiotic behaviour (exclusive bivalent pairing of homologues), which leads to full fertility and disomic inheritance (Feldman and Levy 2005; Griffiths et al. 2006).

Modern studies aiming at genome reconstruction of the bread wheat have shown that as soon as two parental genomes have joined to form an allopolyploid genome, this resulted in a “genomic shock”. Specifically, the issues of distinct genome sizes, chromosome numbers, regulation, and cell cycle progression must have been resolved (Feldman and Levy 2012). Thus, multiple changes must have accompanied the process of genome stabilization (Jones and Hegarty 2009; Tayale and Parisod 2013). To overcome the above conflicts, the two genomes must undergo cytological and genetic diploidization (Feldman and Levy 2005; Ma and Gustafson 2005). As proposed by Feldman and Levy (2005), elimination of DNA sequences along with structural changes in chromosomes is indispensable for cytological diploidization. Gene silencing/gene loss, neofunctionalization, and other epigenetic changes may represent the driving factors of genetic diploidization.

Indeed, for an allopolyploid to form, F_1_ hybrids must first overcome the sterility issue. This issue stems from the lack of homologues in the context of a polyhaploid genome dysregulated genetic control of meiosis and is also attributable to the suppressive effect of the *Ph1* locus on homologous pairing (Sears 1976). Nonetheless, the phenomenon of meiosis in F_1_ hybrids is also associated with the formation of so-called unreduced gametes having somatic chromosome number. Such gametes may unite to form an allopolyploid organism; hence, unreduced gametes were proposed to be involved in the major pathways leading to polyploidy (Bretagnolle and Thompson 1995; Otto and Whitton 2000).

The first cytological studies of the mechanisms underlying the formation of unreduced gametes in intergeneric wheat hybrids (*T. turgidum*, *T. aestivum*) date back to 1930s and are still actively pursued (review Silkova et al. 2011a; Matsuoka et al. 2013; Hao et al. 2014). Considerable research focused on the understanding of cytogenetic mechanisms underlying the formation of the extant bread wheat genome (Xu and Joppa 1995; Matsuoka and Nasuda 2004; Zhang et al. 2007; Cai et al. 2010; Matsuoka et al. 2013; Hao et al. 2014). Chromosome non-disjunction during the first meiotic division (the restitution nucleus forming) is the cytological mechanisms behind the formation of unreduced gametes in triploid F_1_ hybrids between the direct ancestors of allohexaploid bread wheat (*T. aestivum* L., AABBDD genome), *T. turgidum* L. (AABB genome) and *Aegilops tauschii* Coss. (DD genome) (Cai et al. 2010; Matsuoka et al. 2013). The subsequent normal second division produces dyads as the end products of meiosis. This division type was designated as the first-division restitution (FDR) (Xu and Joppa 1995) and called more recently “unreductional meiotic cell division” (UMCD) (Cai et al. 2010). An alternative mechanism for the formation of unreduced gametes in wheat–alien hybrids has also been described, wherein chromosome behaviour is similar to mitosis. Chromosome separation into chromatids at AI and the subsequent omission of the second division and dyads as final products have been demonstrated for the F_1_ of *T. aestivum* L. × *S. cereale* L. (Silkova et al. 2011b), *T. turgidum* L. × *S. cereale* L. (Olesczuk and Lukaszewski 2014), and *T. turgidum* L. × *Ae. tauschii* Coss. (Matsuoka and Nasuda 2004; Zhang et al. 2007, 2008; Hao et al. 2014). This division type was designated as the single division of meiosis (SDM) (Matsuoka and Nasuda 2004). Data by Hao et al. (2014) and Zhang et al. (2007, 2008) showed that both FDR and SDM can result in the formation of functional unreduced gametes in *T. turgidum* × *Ae. tauschii* hybrids. However, the analysis of the mechanisms responsible to SDM and FDR using the refined molecular tools such as centromere pAet6-09 probe, H3Ser10-, CENH3-, and α-tubulin-specific antibodies has not been performed.

Despite recent progress in delineating the underpinnings of meiotic restitution, the mechanisms underlying one-step segregation of sister chromatids remain poorly understood. The key to these mechanisms may lie in the analysis of *mei*-mutants in diploid *Arabidopsis* and *Zea maize* (Consiglio et al. 2004; Brownfield and Köhler 2010; De Storme and Geelen 2013). For over a century, plants have served as an object for studying meiotic chromosome behaviour (Figueroa and Bass 2010). As a result, over 80 genes with meiotic phenotypes have been cloned and characterized in higher plants (Mercier et al. 2015). Most of the aspects related to the hallmarks of meiosis, such as chromosome pairing and recombination, transition from meiosis I to meiosis II, exit from meiosis, and cohesion control have been well described. Amphihaploid plants appear to be poorly suited to study the genetic control of meiosis; however, these hybrids are indispensable for studying polyhaploid meiosis, as they lack the pachytene checkpoint (Li et al. 2009). Careful analysis of meiotic chromosome behaviour in amphihaploids may therefore focus further research on specific meiotic events that contribute to the formation of unreduced gametes, particularly those involved in cell cycle control, spindle assembly, and cohesion.

Earlier, our group reported on the chromosome behaviour in wheat–rye F_1_ hybrids and androgenic haploids, which allowed us to broadly describe the regulation of meiosis in plants with polyhaploid genomes (Silkova et al. 2009, 2011b; Silkova et al. 2013). Our study of meiosis in amphihaploids developed from wheat–rye disomic substitution lines 1R(1A), 1Rv(1A), 2R(2D)_1_, 2R(2D)_2_, 2R(2D)_3_, 5R(5D), 5R(5A), 6R(6A) (2*n* = 42) has provided evidence for a genetic control of chromosome behaviour (Silkova et al. 2011b). In the hybrids studied, several contrasting meiotic phenotypes were observed. About 90 % of meiocytes in hybrids between 2R(2D)_1_ and rye had regular meiosis with random poleward segregation of chromosomes at AI followed by the second division, which consistently yielded tetrads. In hybrids between 1Rv(1A) and 6R(6A) with rye, about 40 % of meiocytes displayed equational distribution of chromosomes and omission of the second division. The hybrids 5R(5D) with rye had high proportions of cells with equationally dividing chromosomes (about 25 % of meiocytes), dyad formation, and partial fertility. In androgenic haploids of the line 6R(6A), half of the meiocytes showed an equational division of the 21 chromosomes present and a failed second division (Silkova et al. 2009). Chromosome behaviour in the meiocytes of androgenic haploids of line 2R(2D)_1_ (Silkova et al. 2009) was similar to that of the hybrids between 2R(2D)_1_ and rye (Silkova et al. 2011b). Sister chromatid segregation during meiosis I combined with the absence of the second meiotic division (i.e. mitotic-like division) was proposed as the mechanism resulting in unreduced gametes (Silkova et al. 2011b).

In the present study, we revisited some of the old questions using modern cytogenetic tools. By directly visualizing the pattern of chromosome segregation and the dynamics of centromere behaviour, we provide evidence arguing in favour of the idea that four distinct chromosome behaviour types exist in the meiosis of wheat–rye amphihaploids. Data on microtubule dynamics and kinetochore architecture in univalent chromosomes indicate that the two meiotic-like chromosome segregation patterns, reductional and equational plus reductional, represent stand-alone division types rather than intermediate stages of meiosis I. In mitotic-like division, all of the events occur in meiosis I. Namely, the robust bipolar spindle is formed, and back-to-back sister kinetochores anchor spindle microtubules. Sister chromatids separate upon one-step cleavage of cohesin along the chromosome arms and at centromeres. Thus, meiosis terminates, and unreduced microspores are formed. The second scenario resulting in unreduced gametes proceeds as follows: (1) the monopolar spindle assembles in meiosis I; (2) chromosome segregation fails; and (3) the bipolar spindle is formed followed by segregation of sister chromatids in meiosis II.

## Materials and methods

### Plant material

This study used the wheat cultivar, *T. aestivum* L. cv. Saratovskaya 29 (cv S29, BBAADD, 2*n* = 42); the rye cultivar, *S. cereale* L. cv. Onokhoiskaya (RR, 2*n* = 14); and wheat–rye F_1_ hybrids (ABDR, 4*x* = 28). The parental plants of wheat–rye hybrids included four disomic single chromosome wheat–rye substitution lines (2*n* = 42): 1Rv(1A) (*T. aestivum* L. cv. Saratovskaya 29/*S. cereale L*. cv. Vyatka) and 5R(5D), 6R(6A) (*T. aestivum* L. cv. Saratovskaya 29/*S. cereale* L. cv. Onokhoiskaya), 2R(2D) (*T. aestivum* L. cv. Saratovskaya 29/Novosibirskaya 67/*S. cereale* L. cv. Onokhoiskaya) (Silkova et al. 2006, 2007). The lines were crossed as female to the diploid rye, hereafter, 2R(2D)xR, 1Rv(1A)xR, 5R(5D)xR, and 6R(6A)xR. Hybrid genotypes have the *Ph* control system. F_1_ hybrids, wheat, and rye plants were grown in the different conditions: in the field of the Institute of Cytology and Genetics located in Novosibirsk (55°01′00″N. 82°55′00″E), Russia (summer 2013, 2014), and under greenhouse conditions during the autumn–winter seasons in 2012, 2013, and 2014 with temperature 24/18 °C day/night and under a day/night cycle of 16/8 h.

### Meiotic chromosome preparation and fluorescence in situ hybridization

For meiotic studies, young spikes at the appropriate stages were fixed in a (3:1) mixture of 96 % ethanol and acetic acid for 24 h and then stored in 70 % ethanol in a refrigerator. Pollen mother cells (PMCs) were stained with and squashed in 3 % acetocarmine. All of the anthers with PMCs at metaphase I–anaphase I and anaphase II–telophase II were analysed. Each anther was analysed individually, assaying all PMCs in each anther.

For fluorescence in situ hybridization (FISH), spikes were fixed in 45 % acetic acid for 2–4 h at room temperature, anthers with meiocytes at MI–AI were selected, squashed, and slides were frozen in liquid nitrogen, dehydrated through a series of alcohols with increasing concentrations of 70, 90, and 96 %, and stored at −20 °C until needed. Each anther was examined individually, and all scorable PMCs were assayed. A total number of 689 meiocytes in 21 plants were examined. For the analysis of mitotic stages, root tips were fixed in a solution of ethanol–acetic acid (3:1, v/v) and stored at −20 °C. Slides were frozen in liquid nitrogen and then cover slips were removed. Slides were dehydrated in a graded series of 70, 90, and 96 % (v/v) ethanol and air-dried.

Centromere structure was analysed using in situ hybridization with a *Ae. tauschii* pAet6-09 probe specific for rye, wheat, rice, and barley centromere repeats (Zhang et al. 2004; Qi et al. 2013). The samples of DNA containing the corresponding repeats were kindly provided by Dr. A. Lukaszewcki (UCR, CA, USA). In situ hybridization with labelled DNA probes was performed according to A. Houben (Houben et al. 2006). Centromere-specific probes were PCR-labelled with digoxygenin 11-dUTP or biotin 16-dUTP. Total DNA from rye was also used as a probe and labelled by nick translation with biotin 16-dUTP or digoxygenin 11-dUTP. Two probes were used separately or in combination (rye DNA/centromere) in different proportions and were mixed with blocking wheat DNA. Chromatin was stained using 1 mg/ml DAPI in Vectashield anti-fade solution (Vector Laboratories).

### Immunofluorescence

Three primary antibodies used were anti-phH3Ser10 (1:1000; Active Motif), which specifically recognized histone H3 phosphorylated at Ser 10; anti-CENH3 (kindly provided by Dr. A. Houben, IPK Gatersleben, Germany, and diluted at 1:850), which specifically recognized the centromeric histone H3 variant; and anti-α-tubulin (Sigma, diluted 1:1000), which detects the α-tubulin of microtubules. The secondary antibodies to anti-phH3S10 and anti-CENH3 were anti-rabbit IgG conjugated with rhodamine (Sigma, diluted 1:100); the secondary antibody to anti-α-tubulin was anti-mouse IgG conjugated with FITC (Sigma, diluted at 1:100).

The method reported by Manzanero et al. (2000) was used with slight variations. Briefly, root tips or anthers were fixed in fresh 8 % paraformaldehyde in PBS for 2 h in a humid chamber, washed 4 × 15 min in phosphate-buffered saline (PBS), and digested at room temperature for 5–15 min in a mixture of 1 % pectinase, 1 % cellulase Onozuka R-10, and 1 % pectolyase Y-23 dissolved in PBS. Root tips or anthers were then washed 3 × 5 min in PBS.

The material was disaggregated on poly-l-lysine-coated slides. After freezing for 15 min at −70 °C and blocking for 30 min in 3 % bovine serum albumin (BSA)/PBS/non-fat milk, incubation with the primary antibodies was completed overnight at 4 °C. Then, slides were washed 4 × 15 min in PBS and incubated with the secondary antibody at room temperature for 1 h. After 4 × 15 min washes in PBS, the slides were counterstained with 4′,6-diamidino-2-phenylindole (DAPI) and mounted in anti-fade Vectashield medium. A total number of 3776 meiocytes (including control wheat and rye plants) in 83 plants were examined.

All slides were examined under an Axio Imager M1 (Karl Zeiss) microscope. Images were recorded with a ProgRes MF camera (Meta Systems, Jenoptic) and processed using the Adobe Photoshop CS2 software.

## Results

Meiosis in 2R(2D)xR is invariably a two-step process unlike that in 1Rv(1A)xR, 5R(5D)xR, and 6R(6A)xR where meiosis is a mixed, one-step and two-step process.

**Fig. 1:**
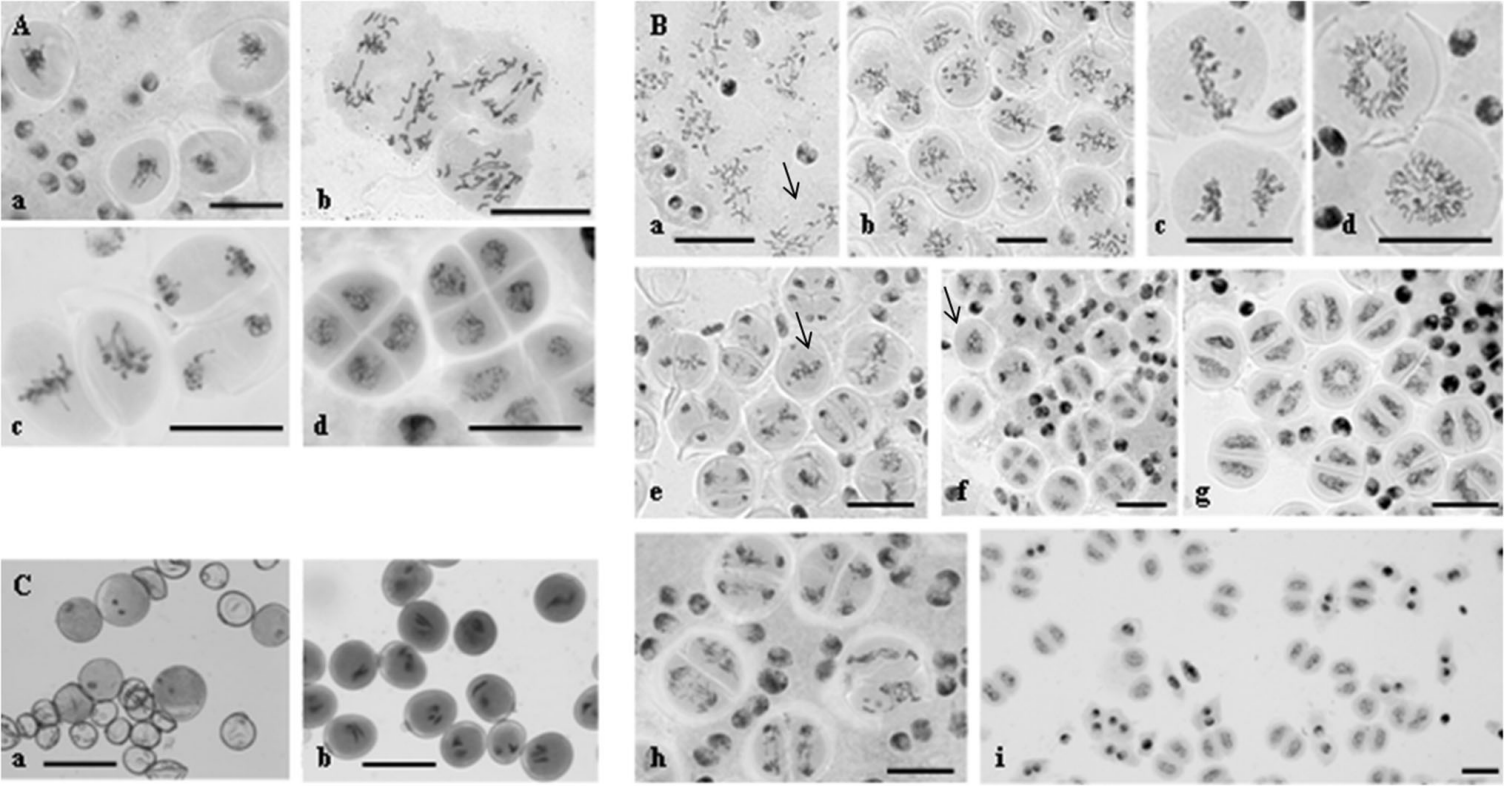
Different types of chromosome behaviour in meiosis of wheat–rye hybrids. **A** Meiosis in 2R(2D)xR hybrids, reductional type of division. **a** Metaphase I–anaphase I, onset of chromosome segregation to the poles. **b** Anaphase I, chromosome segregation. **c** Metaphase II and anaphase II, sister chromatid separation. **d** Telophase II, tetrads are formed. **B** Meiosis in 1Rv(1A)xR, 5R(5D)xR, and 6R(6A)xR hybrids. **a** Reductional (*arrow*) and equational plus reductional division types. **b** Meiocytes with equational division type. **c** Metaphase I and anaphase I, sister chromatid separation. **d** Telophase I, chromosomal ring and chromosomal circle. **e** The second meiosis. Metaphase II and anaphase II, meiocytes with one spindle (*arrow*). **f** Telophase II, meiocytes with restitutive nucleus (*arrow*). **g** Telophase II, dyads and a chromosomal ring. **h** Chromosome missegregation. **i** Unreduced microspores are formed. **C** Pollen grains formed in 1Rv(1A)xR, 5R(5D)xR, 6R(6A)xR hybrids. **a** Sterile and fertile pollen grains. **b** Fertile trinucleate and binucleate pollen grains. *Bars* represent 30 μm

**Fig. 2:**
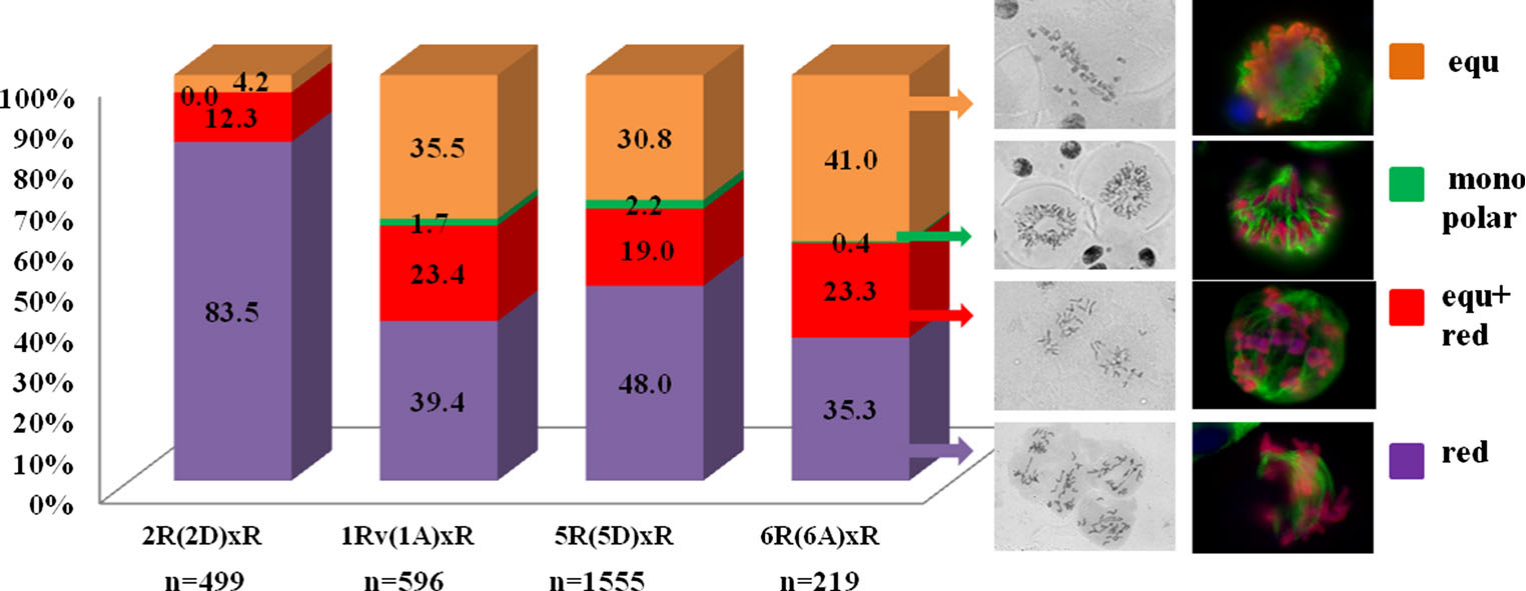
Quantitation of meiocytes (%) displaying distinct chromosomal behaviour in the hybrids 2R(2D)xR, 1Rv(1A)xR, 5R(5D)xR, and 6R(6A)xR. *n* a number of meiocytes were examined

Detailed cytology analysis of meiosis in amphihaploids was performed using acetocarmine staining. In 2R(2D)xR F_1_ hybrids, univalents were observed to be randomly scattered between the poles at metaphase I (MI) (Fig. 1A, b), with 2R2R bivalents segregating as is typical for meiosis. In the second meiotic division, metaphase II (MII) appeared superficially normal following chromosome splitting into sister chromatids at anaphase II (AII) (Fig. 1A, c), but segregation defects were detected (Fig. 1A, c). At telophase II (TII), tetrads with or without micronuclei were formed (Fig. 1A, d), and occasionally microspores of different sizes were produced. Pollen grains were unstained, and plants were sterile. The described meiotic phenotype was repeatedly observed in as much as 80 % of meiocytes regardless of the cultivation conditions (field, greenhouse, and in different growing seasons) (Fig. 2).

**Fig. 3:**
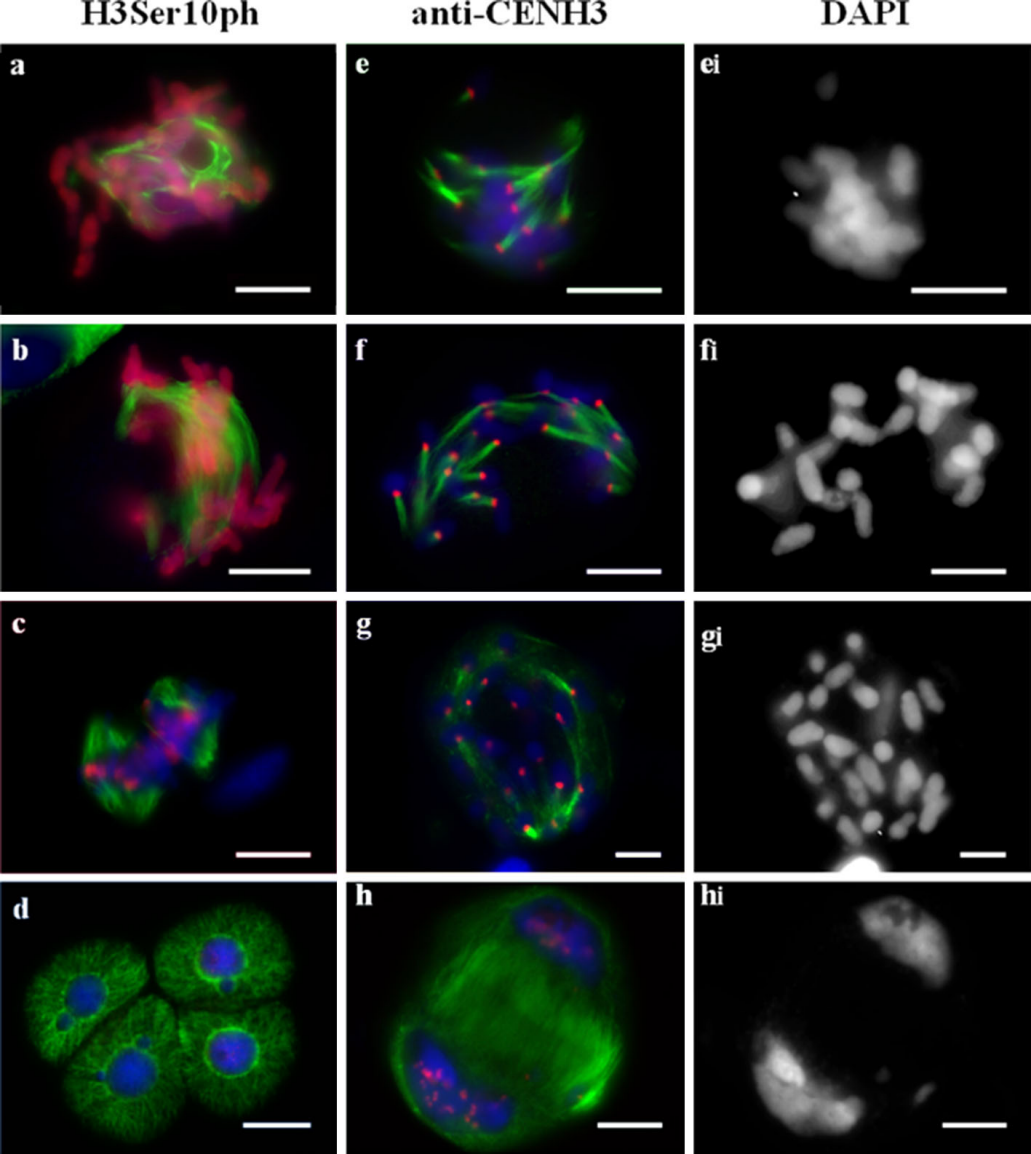
Immunolocalization of H3Ser10ph (**a**–**d**) or CENH3 (**e**–**h**), and α-tubulin in meiosis of 2R(2D)xR hybrids. Reductional type of division. **a** Prometaphase I–metaphase I. **b** Anaphase I, chromosome distribution. **c** Metaphase II. **d** Telophase II. **e** Prometaphase I–metaphase I. **e**_**i**_ The same cell as **e**, DAPI counterstained. **f**, **g** Anaphase I, chromosome distribution. **h** Telophase I. DAPI channel is shown in the *right column* (**e**_**i**_, **f**_**i**_, **g**_**i**_, **h**_**i**_). DNA *blue*, H3Ser10ph and CENH3 labelling *red*, α-tubulin labelling *green*. *Bars* represent 10 μm

The first meiosis in 1Rv(1A)xR, 5R(5D)xR, and 6R(6A)xR hybrids displayed a number of peculiar features (Fig. 1B). Four types of meiocytes were observed. Meiocytes exhibit one of the following chromosomal patterns (Fig. 1B, a, b): (1) random distribution between the poles; (2) some of the chromosomes were scattered, whereas the remainder were located at equator and split into sister chromatids (Fig. 1B, a); (3) all of the chromosomes, except for one or two, exhibited equatorial localization and produced sister chromatids (Fig. 1B, b, c); and (4) chromosomes formed a ring pattern or were located close to each other and formed a circle (Fig. 1B, d). In our earlier study, we defined the pattern exhibiting a random distribution of chromosomes as reductional segregation. The pattern exhibiting equational alignment of chromosomes followed by splitting into sister chromatids was termed equational, and the pattern where some chromosomes were randomly distributed and other chromosomes formed sister chromatids within the same meiocyte was named equational plus reductional (Silkova et al. 2011b).

The ratio of the three types of meiocytes varied. Overall, greater than 1/3 meiocytes in hybrids 1Rv(1A)xR, 5R(5D)xR, and 6R(6A)xR characterized by a separation of sister chromatids in the first meiosis, but a small number meiocytes (from 0.42 to 2.19 %) had chromosomes arranged as a ring or circle (Fig. 2). Cells with chromosomal circles were detectable in 5R(5D) hybrids most frequently (up to 40.62 %).

Similarly, chromosomes followed different patterns during the second meiotic division (Fig. 1B, e–h). MII progressed normally in some anthers. In other anthers, one could simultaneously observe meiocytes stalled at telophase I (TI), meiocytes progressing through AII, and meiocytes with just a single spindle instead of two (Fig. 1B, e, f). In some of these cells, chromosomes displayed a compacted structure, which is typical for MI. In other cells, the chromosomes appeared somewhat decompacted, as is normally observed at MII. Meiocytes also displayed ring decompacted chromosomes (Fig. 1B, g). In some anthers, only dyads were observed. In addition, microspores in these dyads appeared autonomous, suggesting the completion of chromosome division (Fig. 1B, i). Pollen grains were stained (Fig. 1C, b), and amphihaploids were partially fertile.

At TII, a wide range of meiotic phenotypes were observed. Anthers encompassing only tetrads were observed along with anthers exhibiting a mixture of dyads, monads, tetrads, and triads.

### Four types of chromosome behaviour in meiosis of wheat–rye hybrids

Based on the results obtained in meiotic chromosomes of wheat–rye amphihaploids using the centromeric DNA probe pAet6-09 as well as anti-H3Ser10ph, anti-CENH3, and anti-α-tubulin staining, four types of meiocytes differing in the distribution patterns of hybridization signals were distinguished. Given that phosphorylation of histone H3Ser10 residue in plants is cell cycle dependent and related to cohesion maintenance, we used anti-H3Ser10ph as a marker of cohesion upon sister chromatid segregation and to visualize meiotic stages. In the first meiotic division, anti-H3Ser10ph signals were present all over the chromosomes and became restricted to centromeric regions at anaphase II. Joining or separation of sister kinetochores was traced using anti-CENH3 immunostaining.

### Meiotic phenotype with random distribution of chromosomes (reductional type division)

Ascribing the exact stage of division in hybrid meiocytes is a challenging task because chromosome behaviour in these cells is distinct from that observed in normal meiosis. Stages were defined based on the microtubule (MT) dynamics and distribution of anti-H3Ser10ph and anti-CENH3 signals. At prometaphase I, anti-H3Ser10ph signal covered the entire length of the chromosomes (Fig. 3a) and nucleation of MTs began around the chromosomes. Then, MTs interacted with kinetochores (Fig. 3a, e), and no central spindle was observed in metaphase and anaphase (Fig. 3b, f). However, the chromosomes ultimately moved to the poles that were formed via converging kinetochore MTs (Fig. 3f). A monopolar orientation of chromosomes was observed when MTs were clustered on the one side of kinetochore that was detectable as a single dot of the anti-CENH3 signal (Fig. 3e, f). Meiocytes demonstrating pAet6-09 FISH signals that appeared as dense spots at MI were annotated as undergoing reductional division, too (Suppl. Fig. 4f). Such an organization of centromeres in the first meiotic division mirrors the side-by-side positioning of sister kinetochores. One distinctive feature of this pattern is that chromosome arms fail to separate at AI, and no “x”-shaped chromosomes characteristic of the normal meiosis were observed (Fig. 3f_i_).

Rarely, 1–2 lagging chromosomes were observed on the equator. Each had 1–2 CENH3 signals that corresponded to two sister kinetochores, and MT bundles were oriented in opposite directions (Fig. 3f). Double CENH3 signals in telophase chromosomes corresponded to two sister kinetochores, which is indicative of a monopolar-reductional chromosome segregation pattern (Fig. 3h). In the second meiotic division, the chromosomes split into sister chromatids, and anti-H3Ser10ph signals were only retained in the centromeric regions (Fig. 3c). Tetrads appeared indistinguishable from the wild type (Fig. 3d).

Bent spindles are typical for these meiocytes (Fig. 3g).

### Meiocytes with two patterns of chromosome segregation (equational plus reductional type)

**Fig. 4:**
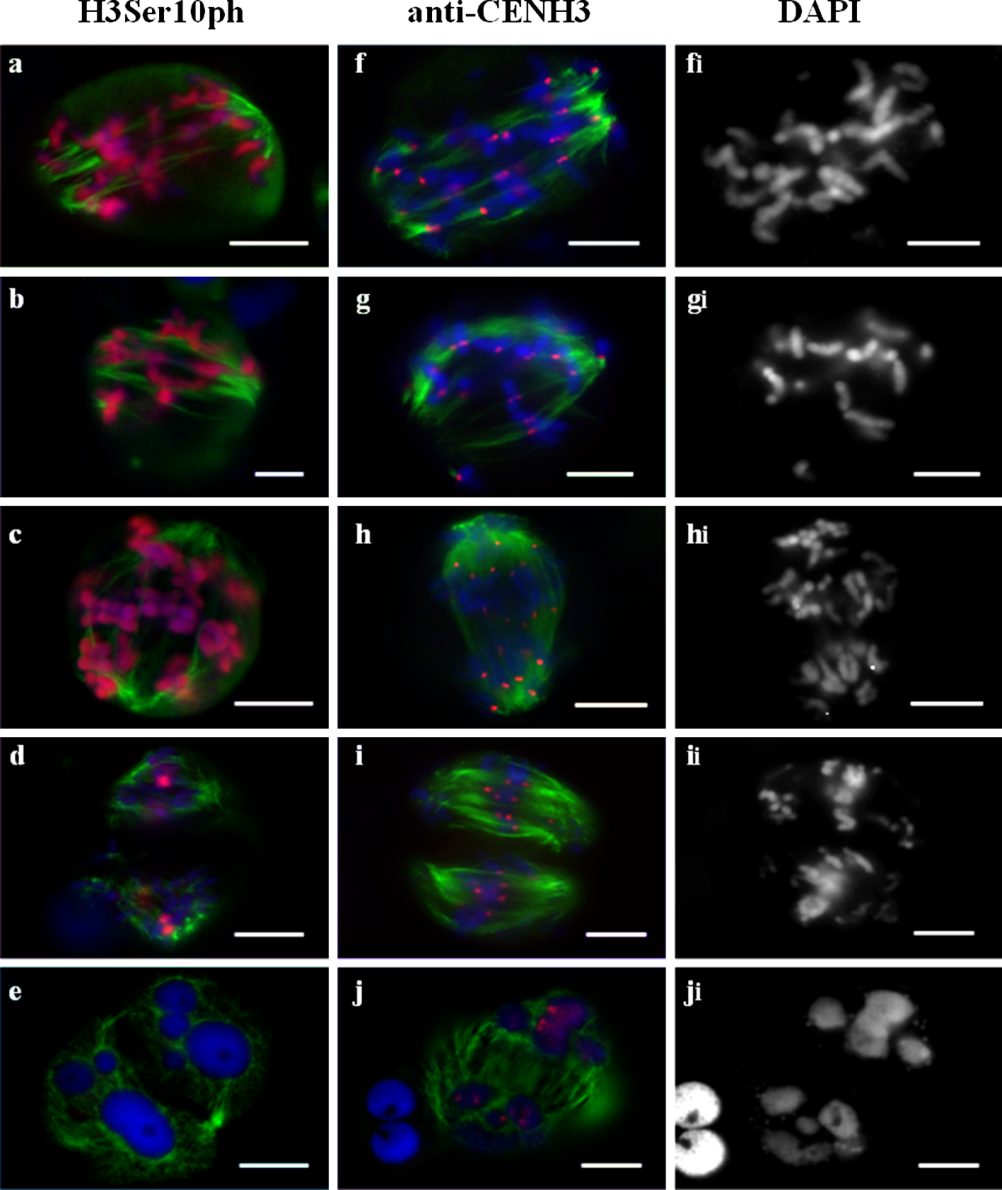
Immunolocalization of H3Ser10ph (**a**–**e**), CENH3 (**f**–**j**), and α-tubulin in meiosis of 1Rv(1A)xR, 5R(5D)xR, and 6R(6A)xR hybrids. Equational plus reductional type of chromosome behaviour. **a**–**c**, **f**, **g** Metaphase I, different meiocytes. **f**_**i**_ The same cell as **f**, DAPI counterstained. **g**_**i**_ The same cell as **g**, DAPI counterstained. **d** Anaphase II. **e** Telophase II. **h** Anaphase I. **h**_**i**_ The same cell as **h**, DAPI counterstained. **i** Anaphase II. **i**_**i**_ The same cell as **i**, DAPI counterstained. **j** Telophase II. **j**_**i**_ The same cell as **j**, DAPI counterstained. DNA *blue*, H3Ser10ph and CENH3 labelling *red*, α-tubulin labelling *green*. *Bars* represent 10 μm

Using anti-CENH3 staining, we observed two types of signals, namely paired spots on chromosomes found at equator and single spots on chromosomes located predominantly near the poles (Fig. 4f, g). This finding indicated that the former type of chromosomes displayed bipolar orientation and appropriately formed sister chromatids, whereas the remainder of the chromosomes were clearly monopolar (Fig. 4g) and either x-shaped or their arms failed to separate (Fig. 4h_i_). Chromosomes undergoing this type of division displayed dense pAet6-09 hybridization spots near the poles. The remainder of the chromosomes lied at the equational plate, and their pAet6-09 hybridization sites appeared as stretched diffuse bands across the chromosomes (Suppl. Fig. 4c). As division proceeded to late anaphase, each of the stretched diffuse sites turned into two independent signals, which was indicative of separated sister centromeres (Suppl. Fig. 4d). In the first meiotic division, anti-H3Ser10ph signal was detected throughout the entire chromosome body (Fig. 4a–c). In these meiocytes, meiotic spindle appeared normal; central spindle and kinetochore fibres were present.

The second meiotic round in these cells was abnormal (Fig. 4d–j). The anti-H3Ser10ph signal mapped to the centromeres of chromosomes that failed to separate during the first division, and no signal was present on sister chromatids (Fig. 4d). Centromeres of sister chromatids were readily stained with anti-CENH3 and formed contacts with spindle MTs (Fig. 4i). Cell division progressed in such cells, ultimately resulting in tetrads with multiple nuclei (Fig. 4e, j). The microspores subsequently formed did not produce fertile pollen.

### Sister chromatid separation in the first meiosis (equational type division)

**Fig. 5:**
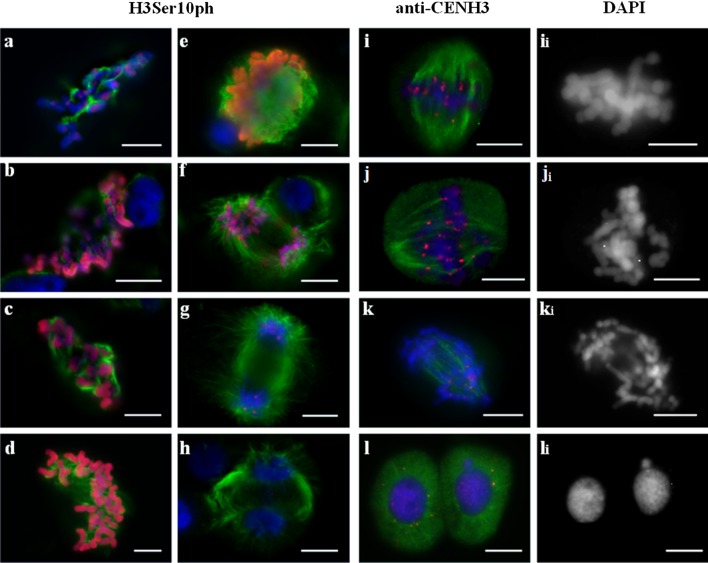
Immunolocalization of H3Ser10ph (**a**–**h**), CENH3 (**i**–**l**), and α-tubulin in meiosis of 1Rv(1A)xR, 5R(5D)xR, and 6R(6A)xR hybrids. Equational type of chromosome behaviour. **a**–**d** Prometaphase I, different meiocytes. **e** Metaphase I. **f** Anaphase I. **g** Late anaphase I. **h** Telophase I. **i**, **j** Metaphase I. **i**_**i**_, **j**_**i**_ The same cell as **i**, **j**, DAPI counterstained. **k** Anaphase I. **k**_**i**_ The same cell as **k**, DAPI counterstained. **l** Telophase I. **l**_**i**_ the same cell as **l**, DAPI counterstained. DNA *blue*, H3Ser10ph and CENH3 labelling *red*, α-tubulin labelling *green*. *Bars* represent 10 μm

Chromosomal behaviour in such meiocytes is essentially mitotic-like. At prometaphase I, MT nucleation occurs near the chromosomes positioned at the equator (Fig. 5a–d). Then, at MI, a bipolar spindle with divergent poles is formed (Fig. 5e). At AI, the chromosomes split to produce chromatids that move polewards (Fig. 5f). At MI, CENH3 signal appears as two separate dots on each of the chromosomes; detection of single CENH3 dots present on telophase groups argues for the presence of separated sister kinetochores (Fig. 5i–k). Stretched diffuse hybridization signals of the centromeric probe pAet6-09 maps to the primary constrictions of chromosomes found at the equational plate at MI (Suppl. Fig. 4a).

**Fig. 6:**
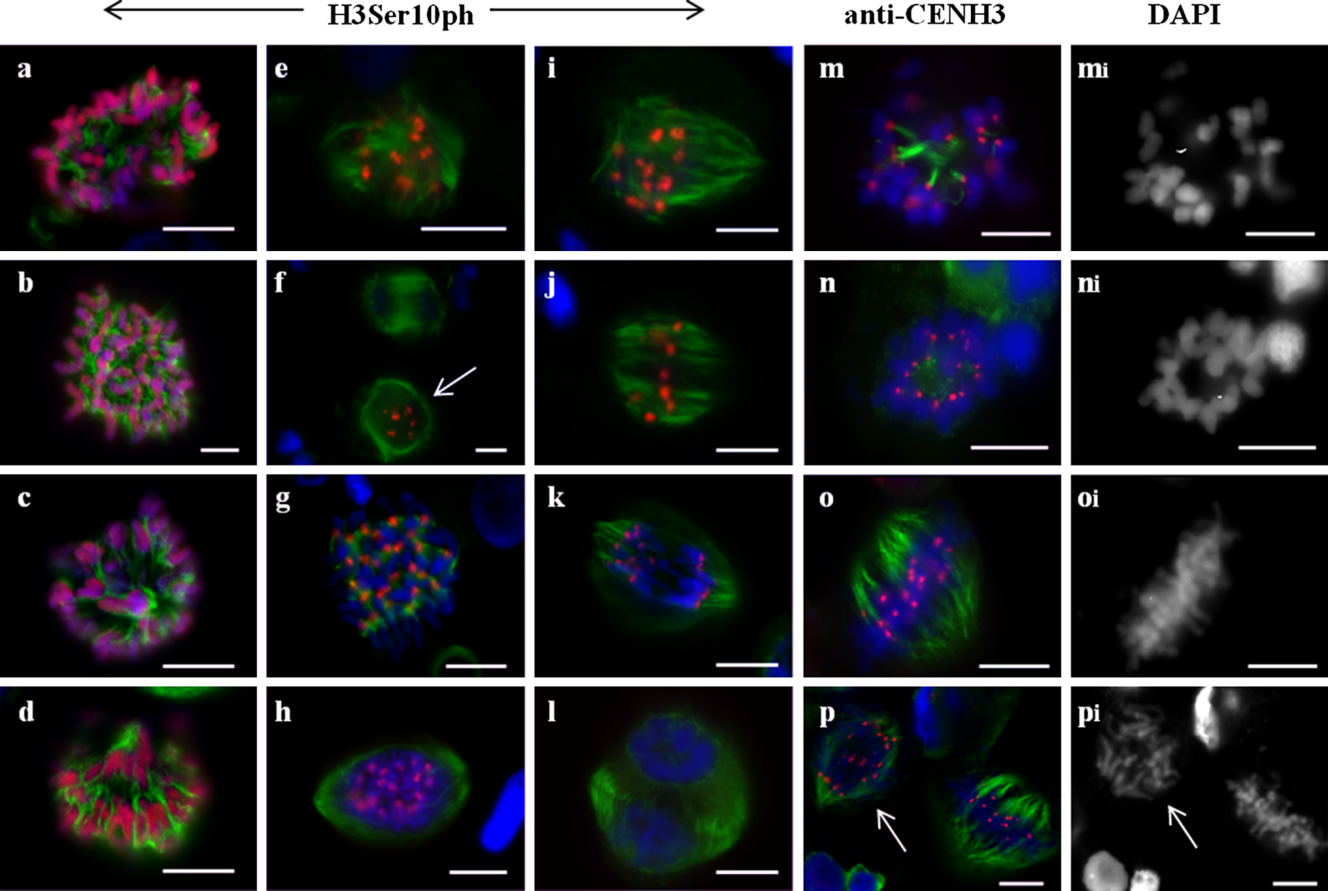
Immunolocalization of H3Ser10ph (**a**–**l**) or CENH3 (**m**–**p**) and α-tubulin in meiosis of 1Rv(1A)xR, 5R(5D)xR, and 6R(6A)xR hybrids. Monopolar spindle formation, blocking of the first division. **a**, **b** Prometaphase I, chromosomal circle (**b**). **c**, **d** Metaphase I, bottom-up view (**c**), top-down view (**d**). **e** Telophase I. **f** Telophase I, restitution nucleus (*arrow*). **g** Prometaphase II. MTs nucleate at kinetochores. **h**, **i** Prometaphase II. Spindle forming. **j** Metaphase II. **k** Anaphase II. **l** Telophase II. **m** Prometaphase I, MT nucleate at kinetochores. **m**_**i**_ The same cell as **m**, DAPI counterstained. **n** Metaphase I, chromosomal ring. **n**_**i**_ The same cell as **n**, DAPI counterstained. **o** Metaphase II. **o**_**i**_ The same cell as **o,** DAPI counterstained. **p** Metaphase II and anaphase II (arrow). **p**_**i**_ The same cell as **p**, DAPI counterstained. DNA *blue*, H3Ser10ph and CENH3 labelling *red*, α-tubulin labelling *green*. *Bars* represent 10 μm

In these cells, the H3Ser10ph signal spanned the entire length of each chromosome (Fig. 5b–e), or, upon transition to AI, the entire chromatid (Fig. 5f). Occasionally, at prometaphase I/metaphase I, the signals appeared brighter at the centromeric regions (Fig. 5a). Later, the H3Ser10ph signals became dimmer and disappeared at TI (Fig. 5g, h). When observing at the cells during the second division, meiocytes that visually corresponded to the interkinesis stage and that lacked any H3Ser10ph or CENH3 signals were noted (Figs. 5h, 6f_l_). We interpreted this phenomenon as the completion of division.

### Blocking of the first division

An exclusive type of chromosome behaviour producing a monopolar spindle was uncovered. Upon nuclear envelope breakdown, the chromosomes were observed tightly clustered together, and MT nucleation at kinetochores began (Fig. 6a, m). Later, the chromosomes remained clustered, albeit to a lesser extent, and bundles of kinetochore MTs that were oriented chaotically were actively formed (Fig. 6b). Next, a hat-shaped group of chromosomes moving in one direction would appear, wherein MT bundles were co-oriented polewards (Fig. 6c, d). A monopolar spindle was thus formed. No separation of chromosomes producing sister chromatids ensued, and the kinetochores did not split (Fig. 6m, n). Localization pattern of the centromeric DNA probe and the CENH3 signals on chromosomes typically appeared as single dots (Suppl. Fig. 4e; Fig. 6n). In rare cases, double FISH signals were observed; however, sister chromatids were never observed. Next, MT became disoriented, and meiocyte progressed through the telophase, which was marked with the anti-H3Ser10ph signal on centromeric regions (Fig. 6e, f). The second meiotic division (centromeric H3Ser10ph staining) began with MT nucleation around the chromosomes (Fig. 6g) followed by the formation of a bipolar spindle (Fig. 6h–j) wherein chromosomes displayed a bipolar orientation of kinetochores (as evidenced by twin CENH3 signals) (Fig. 6o, p). Cell division ended with sister chromatid segregation and the formation of microspores with an unreduced chromosome number (Fig. 6k, l, p).

## Discussion

### Meiotic restitution in hybrids is under genetic control

Our observations indicated that the genotypes of disomic wheat–rye substitution lines contribute to the regulation of meiosis in wheat–rye (ABDR, 4*x* = 28) amphihaploids. The 2R(2D) genotype tends to promote reductional division, which ultimately produces sterile gametes. In contrast, in the genotypes 1Rv(1A), 5R(5D), and 6R(6A) in addition to reductional and equational plus reductional division types, an equational chromosome segregation pattern and monopolar spindle formation are observed. The latter two types of chromosome behaviour result in the formation of unreduced gametes in the hybrids.

It was shown previously that unreduced gametes produced in interspecific/intergeneric hybrids of bread wheat are controlled by the genetic make-up of the parental species (Wagenaar 1968; Xu and Joppa 1995; Matsuoka and Nasuda 2004; Zhang et al. 2007, 2008; Matsuoka et al. 2013; Hao et al. 2014). For instance, *T. turgidum* var. Langdon was demonstrated to cause a high frequency of FDR (the formation of a restitution nucleus in the first division and a normal second division) in hybrids with rye (*S.cereale* L) and *Ae. tauschii* (Xu and Joppa 1995, 2000), yet when hybridized with various accessions of *Ae. tauschii*, various meiotic pathways of meiotic restitution were observed (Matsuoka and Nasuda 2004). Single equational division at anaphase, with dyads as the final meiotic product (SDM), appears to be the major mechanism for the non-reduced gamete formation in F_1_ hybrids between Langdon and *Ae. tauschii* (accession YM9508) line (Matsuoka and Nasuda 2004). To uncover the contribution of *Ae. tauschii* genotypes to the expression of restitution nucleus formation in Langdon—*Ae. tauschii* hybrids, comparative analysis of meiosis in hybrids of var Langdon with various *Ae. tauschii* genotypes was undertaken (Matsuoka et al. 2013). Two representative contrasting accessions of *Ae. tauschii*—one producing fertile hybrids with *T. turgidum* frequently and the other one rarely—allowed mapping of six QTLs that affect the doubling of chromosomes in these hybrids (Matsuoka et al. 2013).

A complete set of 14 Langdon durum D genome disomic substitution lines was used to delineate the genetic components of FDR control in hybrids between rye and *Ae. tauschii* (Xu and Joppa 2000). The results indicated that the chromosome 4A of *T. durum* var Langdon probably carried a gene for high-frequency FDR, whereas 3A and 6A chromosomes likely carried the genes responsible for normal second division of FDR in these crosses. The hybrids of 1D(1A) with *Ae.tauschii* had a high frequency of equational division at the first meiosis. Fertility analysis of F_1_ hybrids between durum wheat (*T. durum* Desf.) Langdon and its 10 disomic substitution lines with *Ae. tauschii* accession AS60 showed increased selfed seedset rates in the hybrids of 1D(1A), 1D(1B), 3D(3A), 4D(4B), 7D(7A), and 2D(2B) with AS60, with lower rates in the hybrids of 3D(3B) + 3BL, 5D(5A) + 5AL, 5D(5B) + 5B, and 6D(6B) + 6BS with AS60 compared with the hybrids of Langdon with AS60 (Zhang et al. 2008). Notably, both FDR and SDM pathways led to unreduced gametes that in turn produced seeds (Zhang et al. 2008).

Recently, a QTL named QTug.sau-3B was identified on the wheat chromosome 3B that was shown to affect hexaploidization of *T. turgidum* × *Ae. tauschii* hybrids (Hao et al. 2014). Comparative genomic analysis indicated that QTug.sau-3B is a collinear homologue of *cyca1;2/tam*, which is known to be responsible for unreduced gamete formation in *Arabidopsis thaliana* (d’Erfurth et al. 2010).

### FDR and SDM coexist in meiosis of partially fertile hybrids

Currently, the cytogenetic mechanism of unreduced gamete formation in amphihaploids is believed to proceed through segregation of sister chromatids of univalents without formation of a restitution nucleus (similar to SDM) and with a restitution nucleus stage (FDR) (Hao et al. 2014). The latter process occurs during TI as a result of non-disjunction of univalents prelocalized at the equatorial plate. Immunostaining experiments using H3Ser10 and α-tubulin antibodies suggested the following mechanism of FDR (Cai et al. 2010; Matsuoka et al. 2013). Assembled during the first meiotic division, the bipolar spindle collapses, and sister chromatids fail to segregate to the poles. Thus, restitution nucleus is formed, and no cytokinesis ensues. Meiosis II progresses normally and produces dyads, the future unreduced gametes. The observation of equational chromosome segregation in the first meiotic division (in fact, the only one) without restitution nucleus stage is not unprecedented (Matsuoka and Nasuda 2004; Zhang et al. 2007, 2008; Silkova et al. 2011b; Hao et al. 2014; Olesczuk and Lukaszewski 2014). Data by Zhang et al. (2007, 2008) showed that both FDR and SDM can result in the formation of functional unreduced gametes in *T. turgidum* × *Ae. tauschii* hybrids.

In the present work, we provide evidence for coexistence of FDR and SDM in the meiosis of partially fertile F_1_ hybrids between 1Rv(1A), 5R(5D), 6R(6A) lines and rye. During SDM, equational segregation of univalents in the first and only meiotic division occurs. Upon FDR, monopolar spindle is formed at meiosis I, which blocks chromosome segregation, and so no cytokinesis stage follows; then, at meiosis II, sister chromatids segregate.

### Segregation of sister chromatids in the first and single division of meiosis

During SDM, double anti-CENH3 signals mark functional sister kinetochores attached to the MTs of opposing spindle poles. Diffuse and “stretched” hybridization signals observed for the centromeric probe pAet6-09 analogous to the pattern observed in mitotic chromosomes and chromosomes undergoing the second meiotic division (Suppl. Fig. 2d, g) are characteristic of univalent chromosomes at MI. Clearly, this mitotic-like structure of chromosomes with back-to-back kinetochore geometry and centromere tension facilitates bi-orientation. MT nucleation begins around the chromosomes located close to the equator. At MI, sister kinetochores attach to microtubules, and bipolar spindle distributes sister chromatids to the poles at AI. The occurrence of these events during the first meiotic division is supported by the localization pattern of H3Ser10ph, when H3Ser10ph signal is distributed across the entire length of chromosomes and becomes restricted to the centromeric regions by the end of AI (Manzanero et al. 2000; Kaszas and Cande 2000).

In plants, distribution of H3Ser10ph correlates with the maintenance and release of sister chromatid cohesion (Manzanero et al. 2000; Kaszas and Cande 2000). During meiosis I, cohesion is maintained throughout the entire length of a chromosome, which matches the observed distribution of H3Ser10ph signal. During mitosis and meiosis II, cohesion is only maintained at pericentromeric regions, as evidenced by H3Ser10ph staining. Taking into account that H3Ser10ph signals entirely cover the chromosomes in amphihaploids (Fig. 5), one can speculate that cohesion complex may keep sister chromatids together during MI. REC8 protein, a meiosis-specific paralog of α-kleisin subunit Scc1, may be imagined to be part of cohesion complex (Watanabe and Nurse 1999). This protein is known to be required for the maintenance of centromeric cohesion at meiosis I (Klein et al. 1999; Watanabe and Nurse 1999). Disruption of AtREC8 was reported to lead to a bipolar orientation of the kinetochores (Chelysheva et al. 2005), whereas *afd1/Zmrec8* mutants displayed deficient synapsis and equational chromosome segregation at AI (Golubovskaya et al. 2006). One of the peculiar features of sister chromatid separation during the first meiotic division in amphihaploids cells—one-step removal of cohesion—was observed in our experiments, but this effect is not attributable to the disruption of REC8. In wild-type meiotic and mitotic MI, the SGO–PP2A complex binds to and dephosphorylates cohesin, thereby protecting centromeric cohesion from separase (Ishiguro et al. 2010; Marston 2015). In the absence of REC8, SGO does not associate with chromatin (Hamant et al. 2005). One can therefore expect that SGO loading does occur in amphihaploids, but no REC8-mediated protection of cohesin follows. Based on this, one-step cohesion removal becomes possible due to the disruption of the SGO–PP2A complex (in meiosis) or due to sister chromatid segregation in the context of mitosis (Marston 2015).

Formation of dyads representing the end products of microsporogenesis implies that both CDK1 and APC/C activities are completely abolished by the end of the first division. In contrast, in normal meiosis, some residual CDK1 activity is always present (Nasmyth and Haering 2009). Thus, the exit from cell division may be promoted by complete degradation of cyclin, which is characteristic of mitosis but not meiosis. Notably, mutation of a homologue of *cyca1;2/tam* results in the formation of unreduced gametes in *A. thaliana* (d’Erfurth et al. 2010). This gene is closely located to the identified QTL affecting hexaploidization in wheat (Hao et al. 2014). Apparently, misregulation of cyclin activity also may occur in amphihaploids.

### Monopolar spindle formation in the first meiosis as mechanism leading to FDR

In 1Rv(1A)xR, 5R(5D)xR, and 6R(6A)xR hybrids, one could observe meiocytes with monopolar spindles. Chromosomes were present as univalents with unsplit kinetochores and were not locked into stable positions. Instead, they oscillated towards and away from the single pole. Most of the studies of the mechanisms of monopolar spindle formation and subsequent block of mitotic cell cycle address the down-regulation or absence of kinesin 5 (Kapoor et al. 2000; Kapitein et al. 2005). Similar to other organisms, plant kinesin 5 stabilizes the spindle at equator by fixing the plus ends of MTs (Zhu and Dixit 2012). Inhibition of the Cdc2/CDK1 kinase results in abnormal bipolar mitotic spindle in *Vicia faba* and alfalfa (Binarová et al. 1998). Specifically, the chromosomes in such cells did not line up at the metaphase plane but instead formed a circle with their kinetochores facing the centre and arms oriented outwards. Later, CDK1-cyclin B must phosphorylate kinesin 5 to bind the spindle-associated MTs (Daire and Pous 2011). When endogenous kinesin 5 (Eg5) was replaced with a non-phosphorylatable Eg5T937A-GFP fusion protein in *Xenopus* egg extracts, monopolar spindles were typically formed (Cahu et al. 2008).

In the present work, we did not observe a classic collapse of a bipolar spindle that would correspond to the phenotype of a lack of kinesin 5 function (stabilization issues in the midzone of a bipolar spindle and slippage of half-spindles) (Bannigan et al. 2007). Chromosomes had a common kinetochore, which precluded the assembly of a normal bipolar spindle. MT nucleation began around the chromosomes and interacted with kinetochores. Prometaphase-stage meiocytes displayed disoriented bundles of kinetochore MTs. Subsequently, MT bundles converged to form a single pole. Thus, the hat-shape ensemble of chromosomes resulted from the peculiar MT organization. According to Zhang and Dawe (2011), kinetochores promote microtubule nucleation and form kinetochore fibres by stabilizing the plus ends, whereas the minus ends become aggregated into loose poles by natural bundling factors such as kinesins (Bannigan et al. 2008). This scenario is hard to refute. Nevertheless, based on the presently available data, the mechanisms behind the monopolar orientation and focusing of MT bundles remain speculative.

### Prolonged meiosis I or distinct mechanisms of chromosome behavioural control?

In *T. turgidum* × *Ae. tauschii* hybrids, the cytological mechanism of meiotic restitution involves prolonged cell division during meiosis I, disassembly of a bipolar spindle, nuclear restitution stage, re-assembly of a bipolar spindle, and segregation of sister chromatids in AII (Matsuoka et al. 2013; Hao et al. 2014). Our analysis indicates that wheat–rye hybrids lack a prolonged cell division during meiosis I. Instead, they display two basic types of meiocyte formation, reductional division and equation plus reductional division. During the course of reductional division, sister chromatids are randomly distributed in two groups. APC/C likely does not function properly because no spindle checkpoint occurs. Kinetochores fail to simultaneously establish contacts with opposing poles (monopolar orientation of CENH3 and pAet06-09 dots), which is likely due to the peculiar organization of the centromeric region. Side-by-side geometry of centromeric region results in the failure to establish a classic central spindle; thus, a functional bipolar spindle is not formed.

Meiocytes undergoing equational plus reductional division display monopolar orientation of kinetochores and MT bundles (reductional chromosome segregation) and a bipolar orientation of sister kinetochores attached to the bipolar spindle MTs. Chromosomes located at the equator divide and form sister chromatids that segregate to the poles where they join the chromosomes with monopolar orientation. Equatorial convergence of all chromosomes was never observed in such meiocytes, and a phragmoplast was formed upon segregation of sister chromatids.

Thus, in wheat–rye amphihaploids, two distinct types of division are substituted for the prolonged cell division at MI. The stage that is described as a prometaphase in *T. turgidum* × *Ae. tauschii* hybrids corresponds to the reductional segregation in *T. aestivum* × *S. cereale* hybrids, whereas equatorial travelling of chromosomes as their “kinetochores switch from monopolar to bipolar orientation” in *T. turgidum* × *Ae. tauschii* (Matsuoka et al. 2013) is best described as equational plus reductional segregation in *T. aestivum* × *S. cereale* hybrids.

## Conclusion

**Fig. 7:**
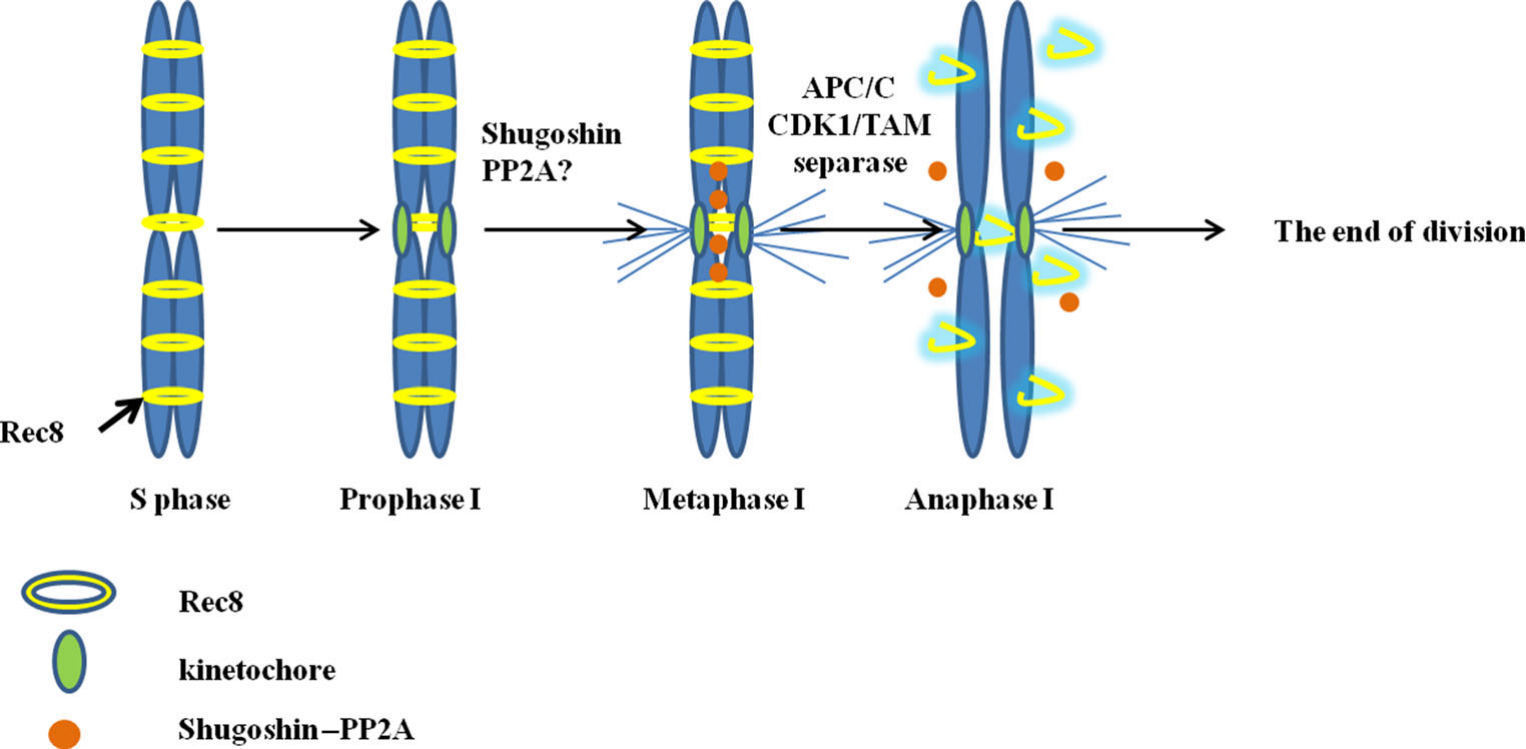
Cartoon of the events during the mitotic-like division in the wheat–rye amphihaploids. During the S phase, Rec8 cohesin becomes loaded onto the chromosomes, thereby joining sister chromatids. In the late prophase (end of pachytene, early diplotene), cyclin CYCA1;2 associates with CDK1; a kinetochore is formed. Metaphase I, CYCA1;2–CDKA complex activity peaks; equatorial positioning of chromosomes, sister chromatids are kept together by cohesion, and kinetochores assume back-to-back geometry and are attached to the opposite poles of the spindle. APC/C^cdc20^ becomes activated to degrade securin; separase activates and cleaves cohesin rings. Cohesin is removed from both the chromosome arms and the centromeres. Consequently, SGO1–PP2A complex functions abnormally during the meiotic program. Univalents split to form sister chromatids that segregate to the poles. Anaphase I, activity of APC/C is so high that it completely degrades cyclin, and CDK1 kinase activity decreases. Division exits from meiosis

Our analysis is indeed limited by the methods available to us. Thus, it is difficult to unambiguously establish the mechanisms underlying formation of unreduced microspores in wheat–rye amphihaploids. However, based on the current understanding of cell cycle regulation, we describe a sequence of molecular events that may underlie the observed one-step separation of sister chromatids (Fig. 7).

Our results indicate that there are two distinct mechanisms of how unreduced gametes are formed in the meiosis of wheat–rye hybrids. One is sister chromatid segregation in the first and only meiotic division (SDM). The other mechanism involves formation of a monopolar spindle in the first meiotic division followed by the segregation of sister chromatids during the second meiotic division (FDR).

Overall, these chromosome behaviours should not be taken as strict abnormal, as the meiotic modifications observed are perfectly compatible with each other and are genetically controlled in wheat–rye hybrids. In our opinion, this adds yet another piece to the puzzle of meiotic control in partially fertile amphihaploids.

### **Author contribution statement**

 OS conceived of the study, designed, performed experiments, analysed, interpreted data, and wrote the manuscript; DL performed experiments, interpreted data, and contributed to Figs. 3–6.

## Electronic supplementary material

Below is the link to the electronic supplementary material.

Supplementary material 1 (PDF 334 kb)

Supplementary material 2 (AVI 30558 kb)

Supplementary material 3 (AVI 35114 kb)

## References

[CR1] Adams KL, Wendel JF (2005). Polyploidy and genome evolution in plants. Curr Opin Plant Biol.

[CR2] Bannigan A, Scheible W-R, Lukowitz W, Fagerstrom C, Wadsworth P, Somerville C, Baskin TI (2007). A conserved role for kinesin-5 in plant mitosis. J Cell Sci.

[CR3] Bannigan A, Lizotte-Waniewski M, Riley M, Baskin TI (2008). Emerging molecular mechanisms that power and regulate the anastral mitotic spindle of flowering plants. Cell Motil Cytoskelet.

[CR4] Binarová P, Dolezel J, Draber P, Heberle-Bors E, Strnad M, Bögre L (1998). Treatment of *Vicia faba* root tip cells with specific inhibitors to cyclin dependent kinases leads to abnormal spindle formation. Plant J.

[CR5] Bretagnolle F, Thompson J (1995). Gametes with the stomatic chromosome number: mechanisms of their formation and role in the evolution of autopolypoid plants. New Phytol.

[CR6] Brownfield L, Köhler C (2010). Unreduced gamete formation in plants: mechanisms and prospects. J Exp Bot.

[CR7] Cahu J, Olichon A, Hentrich C, Schek H, Drinjakovic J, Zhang C, Doherty-Kirby A, Lajoie G, Surrey T (2008). Phosphorylation by Cdk1 increases the binding of Eg5 to microtubules in vitro and in *Xenopus* egg extract spindles. PLoS One.

[CR8] Cai X, Xu SS, Zhu X (2010). Mechanism of haploidy-dependent unreductional meiotic cell division in polyploid wheat. Chromosoma.

[CR9] Chelysheva L, Diallo S, Vezon D, Gendrot G, Vrielynck N, Belcram K, Rocques N, Marquez-Lema A, Bhatt AM, Horlow C, Mercier R, Mézard C, Grelon M (2005). AtREC8 and AtSCC3 are essential to the monopolar orientation of the kinetochores during meiosis. J Cell Sci.

[CR10] Consiglio F, Carputo D, Monti L, Conicella C (2004). Exploitation of genes affecting meiotic non-reduction and nuclear restitution: arabidopsis as a model?. Sex Plant Reprod.

[CR11] d’Erfurth I, Cromer L, Jolivet S, Girard C, Horlow C, Sun Y, To JPC, Berchowitz LE, Copenhaver GP, Mercier R (2010). The cyclin-A CYCA1;2/TAM is required for the meiosis I to meiosis II transition and cooperates with OSD1 for the prophase to first meiotic division transition. PLoS Genet.

[CR12] Daire V, Pous C (2011). Kinesins and protein kinases: key players in the regulation of microtubule dynamics and organization. Arch Biochem Biophys.

[CR13] De Storme N, Geelen D (2013). Sexual polyploidization in plants-cytological mechanisms and molecular regulation. New Phytol.

[CR14] Estep MC, McKain MR, Diaz DV, Zhong J, Hodge JG, Hodkinson TR, Layton DJ, Malcomber ST, Pasquet R, Kellogg EA (2014). Allopolyploidy, diversification, and the Miocene grassland expansion. PNAS.

[CR15] Feldman M, Levy AA (2005). Allopolyploidy—a shaping force in the evolution of wheat genomes. Cytogenet Genome Res.

[CR16] Feldman M, Levy AA (2012). Genome evolution due to allopolyploidization in wheat. Genetics.

[CR17] Figueroa DM, Bass HW (2010). A historical and modern perspective on plant cytogenetics. Brief Funct Genomics.

[CR18] Golubovskaya IN, Hamant O, Timofejeva L, Wang C-JR, Braun D, Meeley R, Cande WZ (2006). Alleles of *afd1* dissect REC8 functions during meiotic prophase I. J Cell Sci.

[CR19] Griffiths S, Sharp R, Foote TN, Bertin I, Wanous M, Reader S, Colas I, Moore G (2006). Molecular characterization of *Ph1* as a major chromosome pairing locus in polyploid wheat. Nature.

[CR20] Hamant O, Golubovskaya IN, Meeley R, Fiume E, Timofejeva L, Schleiffer A, Nasmyth K, Cande WZ (2005). A REC8-dependent plant Shugoshin is required for maintenance of centromeric cohesion during meiosis and has no mitotic functions. Curr Biol.

[CR21] Hao M, Luo J, Zeng D, Zhang L, Ning S, Yuan Z, Yan Z, Zhang H, Zheng Y, Feuillet C, Choulet F, Yen Y, Zhang L, Liu D (2014). QTug.sau-3B is a major quantitative trait locus for wheat hexaploidization. G3 (Bethesda).

[CR22] Houben A, Orford SJ, Timmis JN, Darby IA, Hewitson TD (2006). In situ hybridization to plant tissues and chromosomes. Methods in molecular biology, V. 326: In situ hybridization protocols.

[CR23] International Wheat Genome Sequencing Consortium (2014). A chromosome-based draft sequence of the hexaploid bread wheat genome. Science.

[CR24] Ishiguro T, Tanaka K, Sakuno T, Watanabe Y (2010). Shugoshin-PP2A counteracts casein-kinase-1-dependent cleavage of Rec8 by separase. Nat Cell Biol.

[CR25] Jones RN, Hegarty M (2009). Order out of chaos in the hybrid plant nucleus. Cytogenet Genome Res.

[CR26] Kapitein LC, Peterman EJ, Kwok BH, Kim JH, Kapoor TM, Schmidt CF (2005). The bipolar mitotic kinesin Eg5 moves on both microtubules that it crosslinks. Nature.

[CR27] Kapoor TM, Mayer TU, Coughlin ML, Mitchison TJ (2000). Probing spindle assembly mechanisms with monastrol, a small molecule inhibitor of the mitotic kinesin, Eg5. J Cell Biol.

[CR28] Kaszas E, Cande WZ (2000). Phosphorylation of histone H3 is correlated with changes in the maintenance of sister chromatid cohesion during meiosis in maize, rather than the condensation of the chromatin. J Cell Sci.

[CR29] Klein F, Mahr P, Galova M, Buonomo SB, Michaelis C, Nairz K, Nasmyth K (1999). A central role for cohesins in sister chromatid cohesion, formation of axial elements, and recombination during yeast meiosis. Cell.

[CR30] Li XC, Barringer BC, Barbash DA (2009). The pachytene checkpoint and its relationship to evolutionary patterns of polyploidization and hybrid sterility. Heredity.

[CR31] Ma X-F, Gustafson JP (2005). Genome evolution of allopolyploids: a process of cytological and genetic diploidization. Cytogenet Genome Res.

[CR32] Manzanero S, Arana P, Puertas MJ, Houben A (2000). The chromosomal distribution of phosphorylated histone H3 differs between plants and animals at meiosis. Chromosoma.

[CR33] Marcussen T, Sandve SR, Heier L, Spannagl M, Pfeifer M, Jakobsen KS, Wulff BBH, Steuernagel B, Mayer KFX, Olsen OA, IWGSC (2014). Ancient hybridizations among the ancestral genomes of bread wheat. Science.

[CR34] Marston AL (2015). Shugoshins: tension-sensitive pericentromeric adaptors safeguarding chromosome segregation. Mol Cell Biol.

[CR35] Matsuoka Y, Nasuda S (2004). Durum wheat as candidate for the unknown female progenitor of bread wheat: an empirical study with a highly fertile F_1_ hybrid with *Aegilops tauschii* Coss. Theor Appl Genet.

[CR36] Matsuoka Y, Nasuda S, Ashida Y, Nitta M, Tsujimoto H, Takumi S, Kawahara T (2013). Genetic basis for spontaneous hybrid genome doubling during allopolyploid speciation of common wheat shown by natural variation analyses of the paternal species. PLoS One.

[CR37] Mercier R, Mézard C, Jenczewski E, Macaisne N, Grelon M (2015). The molecular biology of meiosis in plants. Annu Rev Plant Biol.

[CR38] Nasmyth K, Haering CH (2009). Cohesin: its roles and mechanisms. Annu Rev Genet.

[CR39] Olesczuk S, Lukaszewski AJ (2014). The origin of unusual chromosome constitutions among newly formed allopolyploids. Am J Bot.

[CR40] Otto S (2007). The evolutionary consequences of polyploidy. Cell.

[CR41] Otto S, Whitton J (2000). Polyploid incidence and evolution. Annu Rev Genet.

[CR42] Petersen G, Seberg O, Yde M, Berthelsen K (2006). Phylogenetic relationships of Triticum and Aegilops and evidence for the origin of the A, B, and D genomes of common wheat (*Triticum aestivum*). Mol Phylogenet Evol.

[CR43] Qi LL, Wu JJ, Friebe B, Qian C, Gu YQ, Fu DL, Gill BS (2013). Sequence organization and evolutionary dynamics of *Brachypodium* specific centromere retrotransposons. Chromosome Res.

[CR44] Ramsey J, Schemske DW (1998). Pathways, mechanisms and rates of polyploid formation in flowering plants. Annu Rev Ecol Evol Syst.

[CR45] Ramsey J, Schemske DW (2002). Neopolyploidy in flowering plants. Annu Rev Ecol Evol Syst.

[CR46] Sears ER (1976). Genetic control of chromosome pairing in wheat. Annu Rev Genet.

[CR47] Silkova OG, Dobrovolskaya OB, Dubovets NI, Adonina IG, Kravtsova LA, Roder MS, Salina EA, Shchapova AI, Shumny VK (2006). Production of wheat–rye substitution lines and identification of chromosome composition of karyotypes using C-banding, GISH, and SSR markers. Russ J Genet.

[CR48] Silkova OG, Dobrovolskaya OB, Dubovets NI, Adonina IG, Kravtsova LA, Shchapova AI, Shumny VK (2007). Production of wheat–rye substitution lines based on winter rye cultivars with karyotype identification by means of C-banding, GISH, and SSR markers. Russ J Genet.

[CR49] Silkova OG, Dobrovolskaya OB, Shchapova AI, Shumny VK (2009). Features of the regulation of meiotic restitution in androgenic haploids of wheat–rye substitution lines 2R(2D)1, 2R(2D)3, and 6R(6A) (*Triticum aestivum* L., cultivar Saratovskaya29/*Secale cereale* L., cultivar Onokhoiskaya). Russ J Genet.

[CR50] Silkova OG, Shchapova AI, Shumny VK (2011). Meiotic restitution in amphihaploids in the tribe *Triticeae*. Russ J Genet.

[CR51] Silkova OG, Shchapova AI, Shumny VK (2011). Patterns of meiosis in ABDR amphihaploids depend on the specific type of univalent chromosome division. Euphytica.

[CR52] Silkova OG, Adonina IG, Krivosheina EA, Shchapova AI, Shumny VK (2013). Chromosome pairing in meiosis of partially fertile wheat–rye (ABDR) hybrids. Plant Reprod.

[CR53] Soltis PS, Soltis DE (2009). The role of hybridization in plant speciation. Annu Rev Plant Biol.

[CR54] Soltis DE, Albert VA, Leebens-Mack J, Bell CD, Paterson AH, Zheng C, Sankoff D, de Pamphilis CW, Wall PK, Soltis PS (2009). Polyploidy and angiosperm diversification. Am J Bot.

[CR55] Tayale A, Parisod C (2013). Natural pathways to polyploidy in plants and consequences for genome reorganization. Cytogenet Genome Res.

[CR56] Wagenaar EB (1968). Meiotic restitution and the origin of polyploidy. I. Influence of genotype on polyploid seedset in a *Triticum crassum* × *Triticum turgidum* hybrid. Can J Genet Cytol.

[CR57] Watanabe Y, Nurse P (1999). Cohesin Rec8 is required for reductional chromosome segregation at meiosis. Nature.

[CR58] Wendel JF (2000). Genome evolution in polyploids. Plant Mol Biol.

[CR59] Xu SJ, Joppa LR (1995). Mechanisms and inheritance of first division restitution in hybrids of wheat, rye, and *Aegilops squarrosa*. Genome.

[CR60] Xu SJ, Joppa LR (2000). First division restitution in hybrids of Langdon durum disomic substitution linnes with rye and *Aegilops squarrosa*. Plant Breed.

[CR61] Zhang H, Dawe RK (2011). Mechanisms of plant spindle formation. Chromosome Res.

[CR62] Zhang P, Wanlong L, Fellers J, Friebe B, Gill BS (2004). BAC-FISH in wheat identifies chromosome landmarks consisting of different types of transposable elements. Chromosoma.

[CR63] Zhang L, Yen Y, Zheng Y, Liu D (2007). Meiotic restriction in emmer wheat is controlled by one or more nuclear genes that continue to function in derived line. Sex Plant Reprod.

[CR64] Zhang L, Chen Q, Yuan Z, Xiang Z, Zheng Y, Liu D (2008). Production of aneuhaploid and euhaploid sporocytes by meiotic restitution in fertile hybrids between durum wheat Langdon chromosome substitution lines and *Aegilops tauschii*. J Genet Genomics.

[CR65] Zhu C, Dixit R (2012). Functions of the Arabidopsis kinesin superfamily of microtubule-based motor proteins. Protoplasma.

