# Supplementary material for: Sister chromatid separation and monopolar spindle organization in the first meiosis as two mechanisms of unreduced gametes formation in wheat–rye hybrids
Source: Plant Reprod. 2016 Mar 18;29:199–213. doi: 10.1007/s00497-016-0279-5 (PMC4909807; doi:10.1007/s00497-016-0279-5)
Supplement: Supplementary file 1 — Supplementary material 1 (PDF 334 kb) [file 497_2016_279_MOESM1_ESM.pdf]

Sister chromatid separation and monopolar spindle organization in the first meiosis as two mechanisms of unreduced gametes formation in wheat-rye hybrids

Plant Reproduction

Silkova O.G., Loginova D.B.

Institute of Cytology and Genetics, Siberian Branch of the Russian Academy of Sciences, Lavrentiev ave.10, Novosibirsk, 630090, Russia

RUSSIAN FEDERATION 630090 Novosibirsk, Lavrentyev prospect, 10.

Author for correspondence: Olga Silkova

E-mail: silkova@bionet.nsc.ru

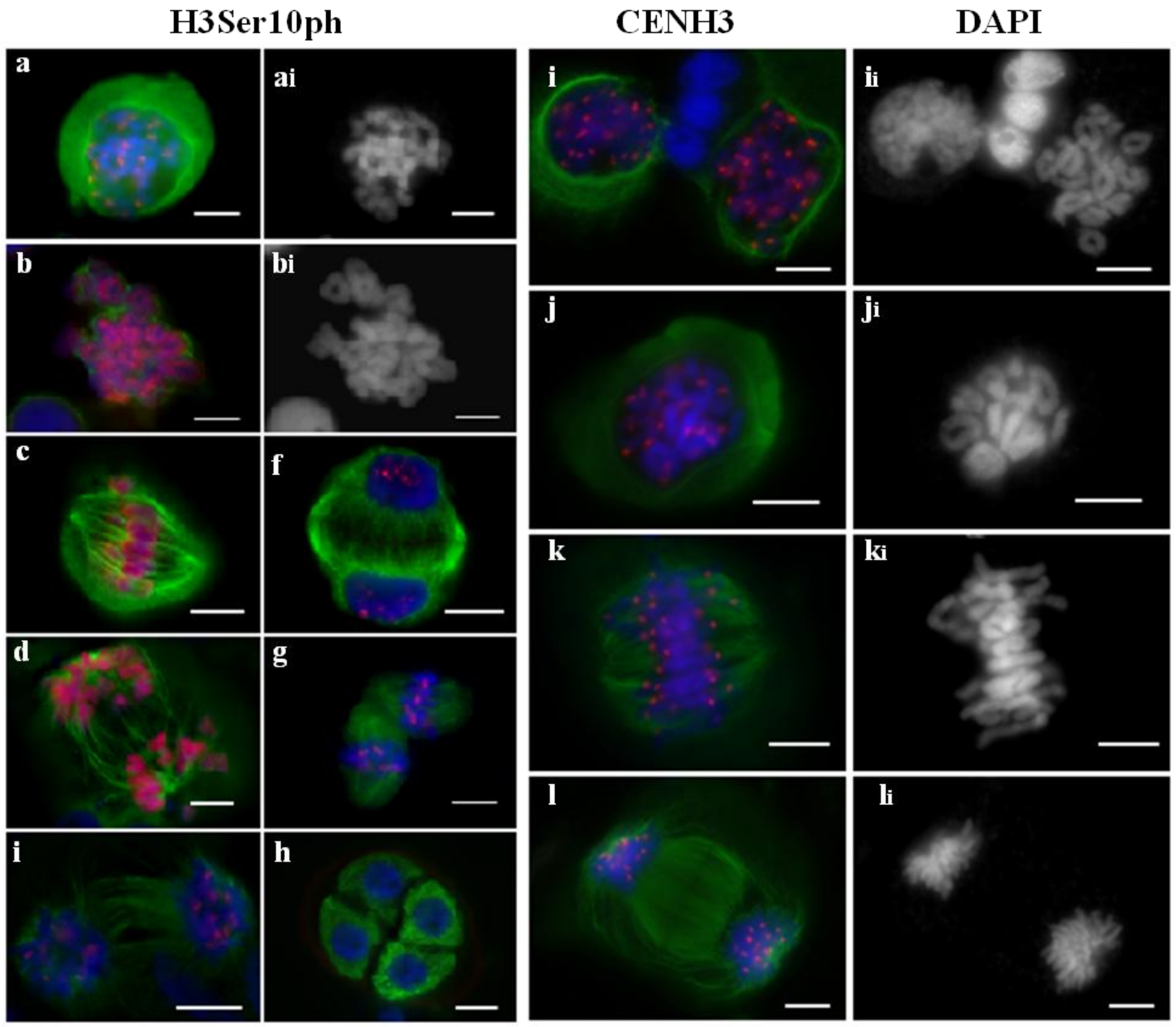

Fig.1 Immunolocalization of H3Ser10ph (**a – h**) or CENH3 (**i – l**) and α-tubulin during meiosis in *Triticum aestivum* L. **a** Diplotene. $\mathbf{a_i}$ The same cell as **a**, DAPI counterstained. **b** Diakinesis. $\mathbf{b_i}$ the same cell as **b**, DAPI counterstained. **c** Metaphase I. **d** Anaphase I. **i** Late anaphase I. **f** Telophase I. **g** Metaphase II. **h** Telophase II. **i** Pachytene and diakinesis. $\mathbf{i_i}$ The same cell as **i**, DAPI counterstained. **j** Diakinesis. $\mathbf{j_i}$The same cell as **i**, DAPI counterstained. **k** Metaphase I. **k l** Telophase I. DNA *blue*, H3Ser10ph and CENH3 labeling *red*, α-tubulin labeling *green*. Bars represent 10 μm

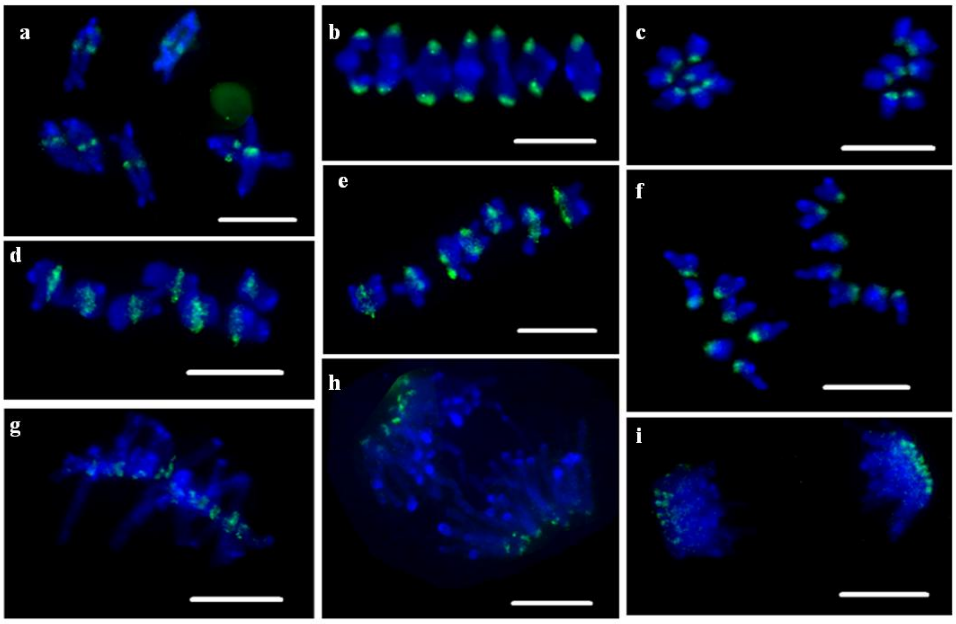

Fig.2 *In situ* hybridization using the centromeric pAet6-09 probe (*green*) to meiotic (**a-f**) and mitotic (**g-i**) chromosomal spreads of rye. **a** Single dense hybridization sites at diplotene. **b** Metaphase I. **c** Anaphase I**. d** Metaphase II, stretched diffuse hybridization sites. **e** Late metaphase II, pAet6-09 signals appear as dense twin spots indicating sister centromere separation. **f** Anaphase II, single dense hybridization sites. **g** Metaphase, stretched diffuse pAet6-09 hybridization sites. Telophase (**h**) and anaphase (**i**), single dense spots of pAet6-09 hybridization sites. Chromatin was counterstained with DAPI (*blue*). Bars represent 10 μm

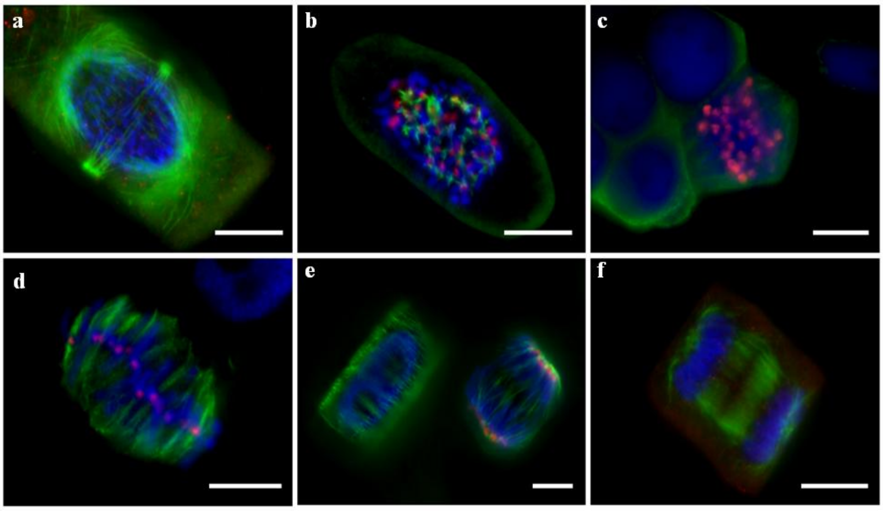

Fig.3 Immunolocalization of H3Ser10ph (*red*) and α-tubulin (*green*) during mitosis in *Triticum aestivum* L. **a** Preprophase band. **b** Prophase. **c** Prometaphase. **d** Metaphase. **e** Anaphase. **f** Telophase. Chromatin was counterstained with DAPI (*blue*). Bars represent 10 μm

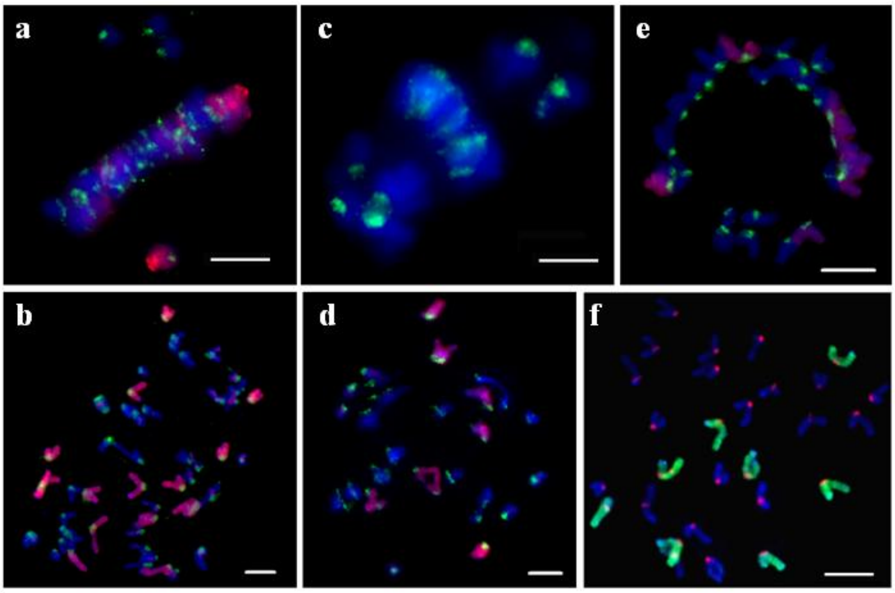

Fig.4 *In situ* hybridization using the centromeric pAet6-09 probe (*green*) and rye genomic DNA probe (*red*) to meiotic chromosomal spreads of wheat-rye amphihaploids. Equational division of chromosomes (**a, b**). **a** Metaphase I, all chromosomes at equatorial plate have stretched diffuse hybridization sites. **b** Anaphase I, separation of sister chromatids**.** Equational+reductional division of chromosomes (**c, d**). **c** Metaphase I, chromosomes at the equatorial plate with stretched diffuse hybridization sites, chromosomes outside of the equatorial plate with single dense hybridization sites. **d** Early anaphase I, sister centromere separation at the equatorial plate, appearance of two local hybridization sites. **e** Chromosomes form a ring with their centromeres facing the center, and arms oriented outwards. **f** Reductional division of chromosomes. The centromeric pAet6-09 probe (*red*) and rye genomic DNA probe (*green*). Metaphase I-anaphase I, chromosomes with single dense hybridization sites. Chromatin was counterstained with DAPI (*blue*). Bars represent 10 μm
